# Adsorption of Mercury on Oxidized Graphenes

**DOI:** 10.3390/nano12173025

**Published:** 2022-08-31

**Authors:** Talia Tene, Stefano Bellucci, Marco Guevara, Fabian Arias Arias, Miguel Ángel Sáez Paguay, John Marcos Quispillo Moyota, Melvin Arias Polanco, Andrea Scarcello, Cristian Vacacela Gomez, Salvatore Straface, Lorenzo S. Caputi, F. Javier Torres

**Affiliations:** 1 Departamento de Química, Universidad Técnica Particular de Loja, Loja 110160, Ecuador; 2 INFN-Laboratori Nazionali di Frascati, Via E. Fermi 54, I-00044 Frascati, RM, Italy; 3 UNICARIBE Research Center, University of Calabria, I-87036 Rende, CS, Italy; 4 Facultad de Recursos Naturales, Escuela Superior Politécnica de Chimborazo (ESPOCH), Coca 220201, Ecuador; 5 Facultad de Ciencias, Escuela Superior Politécnica de Chimborazo (ESPOCH), Riobamba 060155, Ecuador; 6 Instituto Tecnológico de Santo Domingo, Área de Ciencias Básicas y Ambientales, Av. Los Próceres, Santo Domingo 10602, Dominican Republic; 7 Surface Nanoscience Group, Department of Physics, University of Calabria, Via P. Bucci, Cubo 33C, I-87036 Rende, CS, Italy; 8 Department of Environmental Engineering (DIAm) University of Calabria, Via P. Bucci, Cubo 42B, I-87036 Rende, CS, Italy; 9 Grupo de Química Computacional y Teórica (QCT-UR), Facultad de Ciencias Naturales, Universidad del Rosario, Bogotá 111711, Colombia; 10 Grupo de Química Computacional y Teórica (QCT-USFQ), Departamento de Ingeniería Química, Universidad San Francisco de Quito, Diego de Robles y Vía Interoceánica, Quito 17-1200-841, Ecuador

**Keywords:** graphene oxide, reduced graphene oxide, mercury(II), adsorption

## Abstract

Graphene oxide (GO) and its reduced form, reduced graphene oxide (rGO), are among the most predominant graphene derivatives because their unique properties make them efficient adsorbent nanomaterials for water treatment. Although extra-functionalized GO and rGO are customarily employed for the removal of pollutants from aqueous solutions, the adsorption of heavy metals on non-extra-functionalized oxidized graphenes has not been thoroughly studied. Herein, the adsorption of mercury(II) (Hg(II)) on eco-friendly-prepared oxidized graphenes is reported. The work covers the preparation of GO and rGO as well as their characterization. In a further stage, the description of the adsorption mechanism is developed in terms of the kinetics, the associated isotherms, and the thermodynamics of the process. The interaction between Hg(II) and different positions of the oxidized graphene surface is explored by DFT calculations. The study outcomes particularly demonstrate that pristine rGO has better adsorbent properties compared to pristine GO and even other extra-functionalized ones.

## 1. Introduction

It is well known that mercury (Hg(II)) is released into water bodies through the discharge of industrial processes, such as those associated with oil refineries [1], automobile manufacturing [2], and fossil fuel combustion [3]. Hg(II) is established as one of the most dangerous heavy metals for humans and the environment because it is highly toxic, pervasive, cumulative, and persistent [4]. From the human health perspective, Hg(II) ingestion produces detrimental effects such as swelling of the mouth, muscle tremors, and mental disorders [5]. Furthermore, Hg(II) can cause noteworthy menaces to the neurological development of fetuses, newborns, and children [6]. Additionally, mercury consumption in food influences the central nervous system and erethism as well as arrhythmias, cardiomyopathies, and kidney damage. Necrotizing bronchitis and pneumonitis from inhalation of mercury can cause respiratory failure. Mercury is also considered a potent immunostimulant and suppressant. Depending on the exposure dose and individual susceptibility, Hg(II) is capable of producing a series of pathological sequelae, including lymphoproliferation and hypergammaglobulinemia [5,6]. In such a panorama, it is imperative to develop methods for Hg(II) removal. Nowadays, the removal methods include adsorption [7], membrane filtration [8], ion-exchange [9], electrocoagulation [10], and electrodeposition [11]. Among them, adsorption is the most promising technique due to its effortlessness, non-secondary treatment step, and profitable use [12,13,14].

Carbon-based platforms have been suggested as excellent adsorbents for water treatment technologies [15,16]. Among them, activated carbon is the most relevant due to its adaptability in removing different heavy metals. However, various environmental problems have been observed when working with activated carbon because it can be produced from carbonaceous materials by physical or chemical activation or by combining the two processes. The production of activated carbon is divided into four steps: pre-treatment, carbonization, activation, and post-treatment. The most important steps are carbonization and activation, which in turn produce a high level of contamination [17]. More recently, other carbon-based nanomaterials have been proposed for water or wastewater treatment, such as oxidized graphenes, i.e., graphene oxide (GO) and reduced graphene oxide (rGO) [18]. Graphene, a carbon structure organized in a hexagonal honeycomb-like geometry, has received a great deal of interest due to its exceptional electronic, optical, thermal, and mechanical properties [19,20,21,22]. However, the properties of GO and rGO are quite different from those found in graphene since they are covered by oxygen-containing functional groups [23]. The presence of these functional groups provides a hydrophobic feature to oxidized graphenes and interesting adsorption properties [24]. Several top-down approaches have been proposed for preparing graphene and related derivatives. Liquid exfoliation [25,26], shear exfoliation [27], and oxidation-reduction of graphite [28] are some examples. In particular, GO is synthesized by the oxidation of graphite following Hummers or modified Hummers methods [29], which are carried out by treating graphite with H_2_SO_4_, KMnO4, and NaNO_3_. This method has the advantage of large-scale production; however, the generation of toxic gases and residual ions have been observed during the process [30]. To avoid the latter, we have reported elsewhere [31] an eco-friendly protocol for preparing GO with the potential scalability that is important for industrial-scale water treatment. Moreover, we have demonstrated the transformation of GO into rGO by using citric acid (CA), which is found to be an excellent green reducing agent but is not commonly used. 

In recent years, the removal of Hg(II) has been widely explored in extra-functionalized oxidized graphenes [32,33,34,35,36,37] which show an interesting affinity for metal ions [33]. Nevertheless, these types of adsorbents usually show instability in aqueous solutions, reducing the adsorption effectiveness. On the other hand, the involved steps of oxidation, reduction, further functionalization, and final purification cause a bottleneck when using extra-functionalized graphenes for large-scale water treatment. Thus, the removal of Hg(II) in non-extra-functionalized and green-prepared GO or rGO is of vital importance. In this work, such a missing approach is presented, using as-made oxidized graphenes which exhibit interesting adsorption results. The guideline protocol to prepare GO and rGO as well as their morphological, physical, and chemical characterization is also discussed in this study. Finally, the adsorption mechanism is scrutinized in terms of adsorption kinetics, intraparticle diffusion analysis, pH effect, adsorption isotherms, and adsorption thermodynamics. The latter results are complemented by theoretical predictions using density functional theory (DFT) calculations [38,39]. 

## 2. Materials and Method

### 2.1. Materials

All chemical reagents were used without purification. H_2_SO_4_ (Sulfuric acid, 95.0–98.0%, Sigma-Aldrich, St. Louis, MO, USA). KMnO_4_ (Potassium permanganate, ≥99.0%, Sigma-Aldrich). Graphite powder (<150 μm, 99.99%, Sigma-Aldrich). H_2_O_2_ (Hydrogen peroxide, 30%, Merk, Kenilworth, NJ, USA). HCl (Hydrochloric acid, 37%, Sigma-Aldrich). NaOH (Sodium hydroxide, 1310-73-2, 40.00 g/mol, Merk). C_6_H_8_O_7_ (Citric acid, ≥99.5%, Sigma-Aldrich). HgO (Mercury oxide, 21908-53-2, 219.59 g/mol, Merk). 

### 2.2. Synthesis of Oxidized Graphenes 

**Figure 1:**
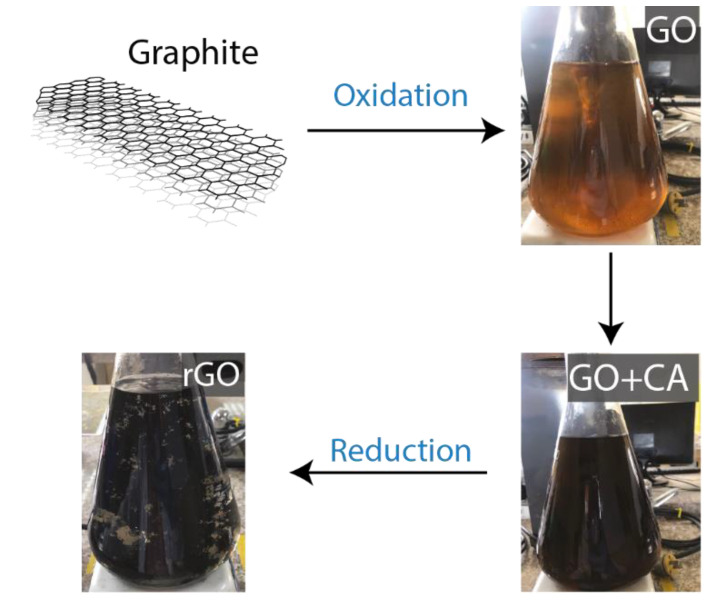
Scheme of the synthesis process of oxidized graphenes, i.e., GO and rGO. The GO+CA suspension acquired a dark color after 20 min of continuous stirring.

Although our procedure has been clearly reported in Ref. [31], a brief description is included (Figure 1) for the sake of completeness of the present study: A borosilicate glass flask was first loaded with powdered graphite (3.0 g), H_2_SO_4_ (70.0 mL), and KMnO_4_ (9.0 g) under careful stirring. The obtained mixture was agitated by adding 150.0 mL distilled water at ∼90 °C, and 500.0 mL distilled water was added, along with 15.0 mL H_2_O_2_. The resultant suspension was collected and washed by centrifugation (at 10,000 rpm and 10 min) with HCl solution and distilled water several times to get a pH close to 6, and then the precipitated material was dried at 80 °C for 2 h to obtain graphite oxide. As an example, 50 mg of dried material was dispersed in 500 mL distilled water by ultrasonication for 15 min. The resultant dispersion was centrifuged to separate the GO flakes from graphite oxide particles. The obtained solution was separated into two parts: one to acquire a well-dispersed GO suspension (which is dried at 80 °C overnight to obtain GO powder), and the other was further treated for preparing rGO. Then, under agitation, 500 mg CA was added to the GO suspension, setting the temperature at 95 °C for 0.5 h. The resultant black precipitates were washed (three times) with distilled water by centrifugation at 5000 rpm for 0.5 h. Finally, the obtained precipitates were dried at 80 °C overnight to obtain rGO powder.

### 2.3. Characterization Techniques 

The following equipment was employed for the characterization of the synthetized materials:

- A scanning electron microscope (SEM, JSM-IT100 InTouchScope) equipped with a JEOL dispersive X-ray spectrometer (EDS) (accelerating voltage of 15 kV) as well as a transmission electron microscope (TEM, JEM 1400 Plus) (operating at 80 kV) were used to observe the surface morphology of GO and rGO.
- Raman spectra of GO and rGO were recorded using a Jasco NRS-500 spectrometer with a 532 nm laser wavelength (0.3 mW, 100X objective).
- Infrared spectra were recorded using a Fourier transform infrared spectrometer (Jasco FT/IR 4000).
- UV-visible measurements were recorded using UV–vis spectroscopy (Thermo Scientific, Waltham, MA, USA, Evolution 220).
- X-ray diffraction measurements were carried out using an X-ray diffractometer (PANalytical Pro X-ray) with a diffraction angle (2*θ*) window of 5–70° and using Cu Kα irradiation with the acceleration voltage of 60 kV and a current of 55 mA.

### 2.4. Adsorption Experiments 

A falcon tube was loaded with a 300 mL aqueous solution of HgO (150 mg L^−1^). At that point, 200.0 mg GO was added to form a suspension. The mixture was stirred at room temperature for 1.0 h. In this phase, the mixture was filtered at intervals through a 0.45 mm membrane filter for all samples, and then the resulting filtrates were examined to determine the remaining Hg(II) content using AAS-cold vapor (standard methods 3112-B; 3111-B.4b). HCl and NaOH 0.1 N were used for setting the pH of the solution (pH = 6.4). To study the effect of pH, the hydronium concentration was varied with HCl and NaOH, and GO was instantly added. The same procedure and experimental conditions were carried out for the adsorption of Hg(II) on rGO.

### 2.5. Gas-Phase Calculations 

DFT calculations, as implemented in the GAUSSIAN16 suite of programs [38], were used to study the theoretical adsorption of the Hg atom onto different models of oxidized graphene surfaces. The ω-B97XD functional was adopted together with all-electron Pople’s split-valence 6-311G(d,p) basis set [40] as the level of theory. The Hg atom was described by adopting the LanL2DZ pseudopotential [41]. The rGO surface is modeled using a molecular model derived from a 6 × 6 × 1 graphene supercell covered with two oxygen functional groups (i.e., hydroxyl and epoxy groups). The boundaries of the models were correctly saturated with H atoms. It is important to note that these gas-phase computations adequately allow introducing a +2 charge into the system. 

## 3. Results and Discussion

### 3.1. Characterization of Oxidized Graphenes 

**Figure 2:**
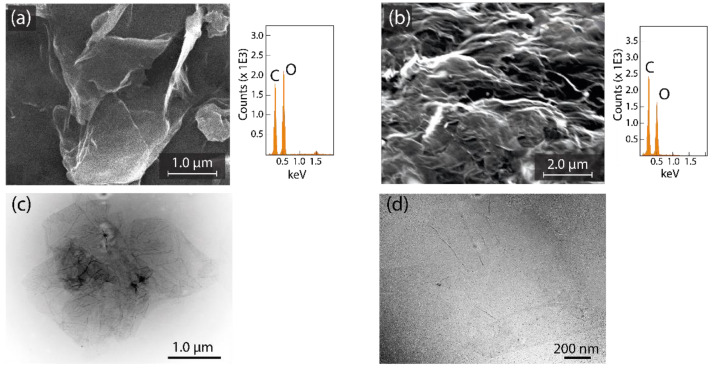
SEM images and EDS measurements of (**a**) GO and (**b**) rGO. TEM images of (**c**) GO and (**d**) rGO.

Figure 2 displays the representative SEM images and EDS results of GO (Figure 2a) and rGO (Figure 2b). GO is characterized by a face-to-face stacking of flakes with several folds and wrinkles on the surface. The elemental analysis shows a C content of 49.7% and an O content of 50.3% (Figure 2a, right panel). On the other hand, rGO shows a highly distorted structure with the formation of mesopores and micropores, which, in turn, is expected to prevent the re-stacking of flakes after the reduction process. The elemental analysis of rGO is C: 62.9%, O: 37.1% (Figure 2b, right panel), which is in agreement with the elimination of oxygen functionalizations as a result of the reduction process.

Figure 2 also shows the representative TEM images of GO (Figure 2c) and rGO (Figure 2d). Several thin sheets with slight folds and wrinkles on the surface and edges are observed in GO, suggesting non-critical damage to its structure derived by the chemical treatment. The dark regions can be ascribed to a high density of *sp*^3^ hybridized zones. After reduction, rGO shows well-defined nanosheets with a regular surface and unfolded edges. The undetected dark zones allow the assumption that *sp*^2^ hybridization has recovered. The semitransparent feature observed in both GO and rGO proposes that these sheets seem to be free of impurities, an imperative result for future nanodevices. 

**Figure 3:**
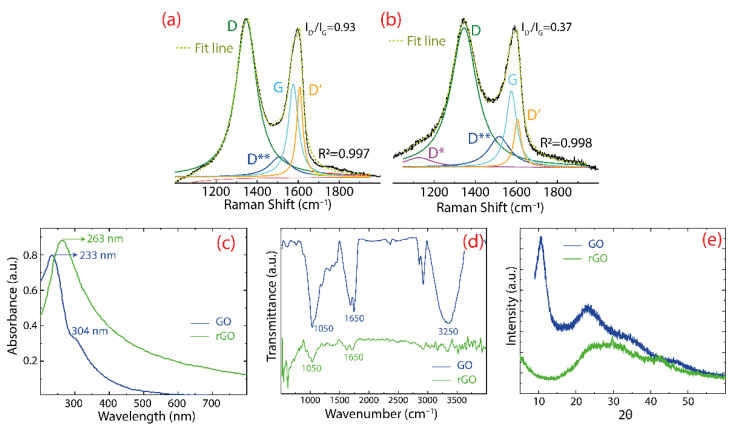
Raman spectra recorded in the 1000–2000 cm^−1^ wavenumber region for (**a**) GO and (**b**) rGO. The intensity was normalized by the D peak and the fitting was made by using Lorentzian functions. Spectroscopic characterization of GO and rGO: (**c**) UV–visible (UV-vis), (**d**) IR spectra, and (**e**) XRD analysis.

Figure 3 displays the Raman spectrum of GO (Figure 3a) and rGO (Figure 3b), whereas Table 1 contains the position and full-width at half maximum (FWHM) of the D, G, D’, D*, and D** peaks. In particular, the D peak is attributed to the basal or edge structural defects [42], while the G peak arises because of the bond stretching of all pairs of sp^2^ hybridized carbon atoms [43]. Through a data fitting phase, other bands are observed in GO: the D** band (1511 cm^−1^) and the D’ band (1608 cm^−1^). The former is a consequence of C–H vibrations in hydrogenated carbon or hopping-like defects [44], while the latter appears due to the presence of different defects, for instance, vacancies, impurities, and the change of *sp*^2^ → *sp*^3^ hybridization [45]. Therefore, a decrease in the intensity of the D’ band can be taken as straight evidence of the GO reduction [28]. A similar data trend is observed in rGO; however, two important differences are noted (after reduction): (i) a barely noticeable D* band is detected at 1124 cm^−1^, which can be attributed to the diamond-like carbon phase [46], and, most importantly, (ii) the intensity of the D’ band is attenuated (with an intensity ratio, I_D’_/I_G_, from 0.93 to 0.37), further supporting the change of GO into rGO.

Figure 3 also shows the characterization by UV-vis (Figure 3c), IR (Figure 3d), and XRD (Figure 3e), where it is observed that GO exhibits a peak at 233 nm which is connected with the $\pi -\pi^{*}$ transitions of C=C bonds [47] and a shoulder-like peak at 304 nm connected with the $n-\pi^{*}$ transitions of C=O bonds. After reduction, the main peak shifts at 263 nm, suggesting the recovery of the electronic conjugation of graphene [48]. The presence of different oxygen functional groups is demonstrated by the FT–IR measurements, showing the following characteristic functional groups: C–O at 1050 cm^−1^, C=O at 1650 cm^−1^, and O–H (or water molecules) at 3250 cm^−1^. These functional groups provide a hydrophilic feature in GO with a good dispersibility in water [49]. After reduction, the intensity of the above peaks is significantly attenuated in rGO, suggesting the removal of functional groups. The XRD analysis of GO shows a narrow peak at 2*θ* = 10.9°, which corresponds to a lattice spacing of 0.81 nm, demonstrating the crystallinity change of graphite into GO [50]. The enlarged lattice spacing is a consequence of the intercalation of functional groups or water molecules. After reduction, the peak becomes broader and moves towards angles, 2*θ* = 22.1°, which matches a lattice spacing of 0.39 nm, confirming the elimination of intercalated molecules or functional groups, decreasing the distance between rGO layers.

### 3.2. Adsorption Kinetics: GO vs. rGO

**Figure 4:**
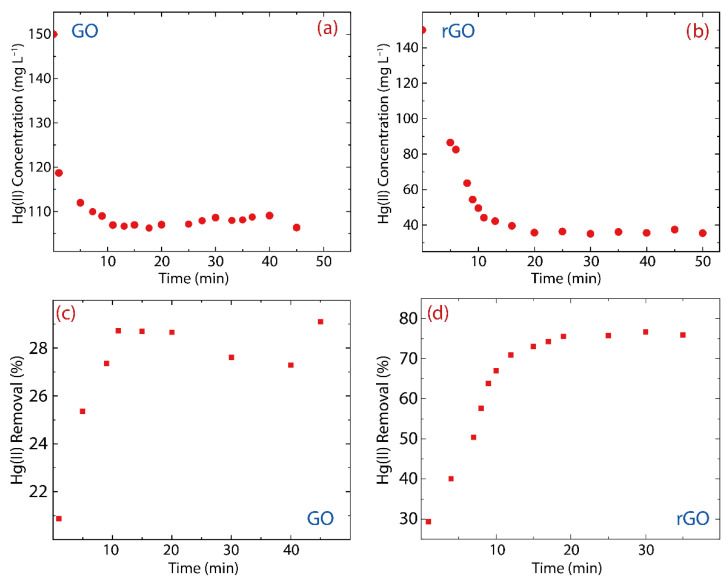
Adsorption kinetics and removal percentage of Hg(II) on (**a**,**c**) GO and (**b**,**d**) rGO under the Hg(II) initial concentration of 150 mg L^−1^.

The adsorption kinetics of Hg(II) on eco-friendly-prepared oxidized graphenes is shown in Figure 4. In order to estimate the effectiveness of as-made adsorbents, GO and rGO were placed in a diluted aqueous solution of HgO. The adsorption capacity ($q_{t}$) can be calculated as follows:

(1) $$q_{t}=\frac{\left(C_{0}-C_{t}\right)\;V}{W}\;$$

where $C_{0}$, $C_{t}$, V, and W are the initial Hg(II) concentration (150 mg L^−1^), the Hg(II) concentration at the time t, the volume of the prepared solution (L), and the adsorbent mass (g), respectively. At the equilibrium, $C_{e}=C_{t}$ and $q_{e}=q_{t}$, which are the so-called equilibrium concentration ($C_{e}$) and the equilibrium adsorption capacity ($q_{e})$. Furthermore, in terms of removal, the effectiveness (RE%) of GO (Figure 4c) and rGO (Figure 4d) can be calculated as:

(2) $$RE\%=\left|\frac{C_{0}-C_{e}\;}{C_{0}}\right|\times 100\;$$

Figure 4 displays the adsorption kinetics of Hg(II) on GO or rGO, considering an interaction time of up to 1.0 h. As observed, GO can rapidly adsorb Hg(II) ions after 10 min, which is defined as the equilibrium adsorption time of GO (Figure 4a). It is worth of noting that its counterpart in rGO is found to be equal to 20 min (Figure 4b). The longer adsorption time observed in rGO can be ascribed to the fact that the rGO structure has more active adsorption sites. 

Regarding the effectiveness, the remaining Hg(II) concentration in the solution treated with GO was found to be ~107 mg L^−1^ (Figure 4a), which means that just 28% of the Hg(II) content was removed (Figure 4c), whereas the remaining Hg(II) concentration in the solution treated with rGO was about ~40 mg L^−1^ (Figure 4b), showing that nearly 75% of Hg(II) content was removed (Figure 4d). The removal percentage using GO ranges from 21% to 29%, showing an increase of 8%, while the removal percentage using rGO ranges from 29% to 77%, which is an increase of 48%, six times more than GO. 

**Figure 5:**
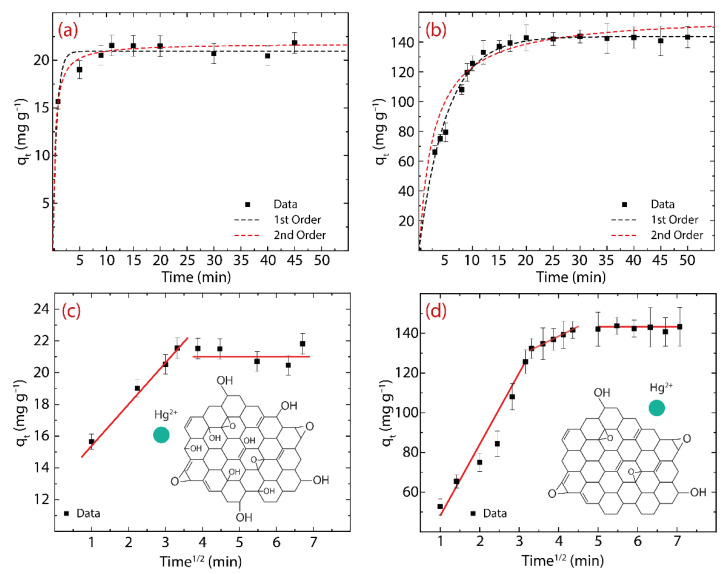
Pseudo-first- and pseudo-second-order models as well as the intraparticle diffusion (IPD) model of the adsorption of Hg(II) on (**a**,**c**) GO and (**b**,**d**) rGO. IPD study shows different regions of linearity. The initial concentration is 150 mg L^−1^.

The adsorption mechanism of Hg(II) on GO or rGO is reported in Figure 5, i.e., the adsorption kinetics (Figure 5a,b) and intraparticle diffusion (IPD) analysis (Figure 5c,d).

The parameters of the pseudo-first-order (PFO) model can be estimated as follows:

PFO(3)dqtdt=k1qe−qt(4)logqe−qt=logqe−k12.303t

where $k_{1}$ represents the PFO rate constant, $q_{t}$ represents the adsorption capacity at a specific time (t), and $q_{e}$ represents the equilibrium adsorption capacity [51]. 

The parameters of the pseudo-second-order (PSO) model can be estimated as follows:

PSO(3)dqtdt=k2qe−qt2(4)tqt=1k2qe2+1qet

where $k_{2}$ denotes the PFO rate constant [51]. The estimated numerical values are reported in Table 2 and related plots in Figure 5a for GO and Figure 5b for rGO.

From the PFO model in GO (Figure 5a), the estimated $q_{e}$ value ($q_{e}=20.9$ mg g^−1^) is similar to the experimentally observed, $q_{e}\left(exp\right)=21.12$ mg g^−1^. Instead, the PSO model slightly overestimates the $q_{e}\left(exp\right)$ value ($q_{e}=21.8$ mg g^−1^). By comparing the metric values, i.e., *R*^2^ and SSE, the adsorption kinetics process is described by the PSO model, suggesting that the adsorption process could be due to chemisorption [52]. In rGO (Figure 5b), the PFO and PSO models show that the estimated values of $q_{e}$ ($q_{e}=143.7$ mg g^−1^ and $q_{e}=151.3$ mg g^−1^, respectively) are slightly above the experimentally obtained values ($q_{e}\left(exp\right)=142.26$ mg g^−1^). The resulting R^2^ and SSE confirm that the adsorption kinetics is best described by the PSO model; that is, the adsorption process in rGO could also be governed by chemisorption.

With the purpose of scrutinizing the gradual diffusion of Hg(II) into the GO or rGO structure, the IPD model can be used as follows:

(7) $$q_{t}=k_{p}t^{0.5}+C\;$$

where $k_{p}$ denotes the IPD rate constant (mg g^−1^ min^1/2^) and intercept C denotes the surface adsorption or boundary layer effect [53]. 

From the theoretical perspective: (i) if C is zero, there is no boundary layer effect and subsequently, the linear line should pass through the origin (which is absent here), and (ii) if C is greater than zero, the contribution of the surface adsorption is larger. The parameters of the IPD model are reported in Table 3, and the plot of uptake capacity vs. the square root of time is shown in Figure 5c for GO and Figure 5d for rGO.

The C values observed in Hg(II) on GO (i.e., C=8.7) and Hg(II) on rGO (i.e., C=44.3) show that a large amount of adsorption arose on the surface, which caused Hg(II) ions to move from the surface to the internal structure of the adsorbent. However, the C value in rGO is approximately five times higher than that found in GO, suggesting that rGO has a larger active adsorption surface, which is likely due to the recovery of *sp^2^* hybridization after the reduction process. 

Interestingly enough, two linear stages are noticed in GO: the early stage shows a faster movement of Hg(II) ions from the aqueous suspension to the GO surface, and the later stage is related to the very slow diffusion of Hg(II) ions throughout the internal GO structure (Figure 5c). Instead, rGO shows an intermediate (extra) region that is ascribed to the steady diffusion of Hg(II) ions from larger pores to smaller pores (Figure 5d). These results confirm that GO does not have enough active sites for capturing Hg(II) ions and therefore its adsorption capacity is reduced. 

In order to further explore the diffusion process, the initial adsorption factor ($R_{i}$) (Table 3) can be computed as follows:

(8) $$R_{i}=\frac{q_{ref}-c}{q_{ref}}\;$$

where c is the ratio of the initial adsorption quantity and $q_{ref}$ is the final adsorption quantity at the longest t. The $R_{i}$ values in GO ($R_{i}=0.59$) and rGO ($R_{i}=0.41$) indicate intermediate initial adsorption and strong initial adsorption, respectively [53]. In particular, the highest adsorption of Hg(II) ions occurs on the rGO surface, confirming that rGO has a vast quantity of active sites for adsorption.

It is worth mentioning that the different oxygen functional groups (such as hydroxyl, epoxy, carboxyl, and carbonyl groups) are randomly distributed in the GO or rGO structure; however, the predominant functional groups on the surface are hydroxyl and epoxy groups. Therefore, inset Figure 5c,d guides the interaction mechanism between Hg(II) and GO or Hg(II) and rGO, which is expected to be mostly electrostatic due to the positive charge of Hg(II) and the negatively charged surface of the adsorbents.

### 3.3. Effect of pH: GO vs. rGO

**Figure 6:**
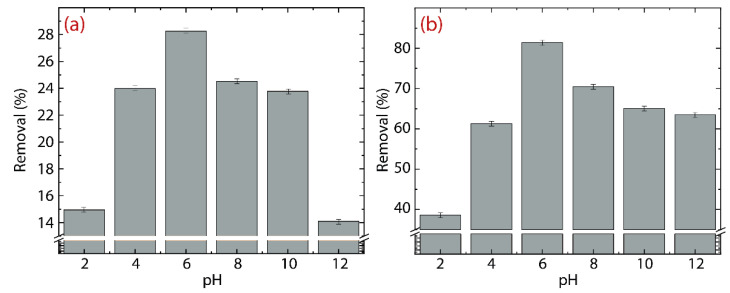
Adsorption of Hg(II) as a function of the initial pH on (**a**) GO and (**b**) rGO. The initial concentration is 150 mg L^−1^.

The consequence of pH on the adsorption of Hg(II) ions is presented in Figure 6a for GO and Figure 6b for rGO. The pH experiment is carried out at 298 K in a range from 2 to 12. It is important to mention that HgO is insoluble in water at pH values > 8; thus, it precipitates and remains in the solution. However, the precipitated HgO could be eliminated by changing the pH to less than 8 to obtain Hg(II) ions that can be easily adsorbed. 

In GO, the removal percentage increases from ~15% at pH =2 up to ~28% at pH =6. After that, the removal percentage decreases from ~24% at pH =8 down to ~13% at pH =12. The drop in the removal effectiveness of Hg(II) at high pH values (>8) can be ascribed precisely to the low solubility of mercury oxide. From pH =4 to pH =8, the average removal percentage is 25.32%. In rGO, the initial removal percentage of ~39% at pH =2 is higher than the value found at the same pH in GO and even higher than the average value. The latter emphasizes the superior adsorbent properties of rGO. A closer inspection shows that the maximum removal percentage of ~80% at pH =6 is 3.3 times greater than the maximum value of removal percentage found in GO. The average removal percentage, from pH =4 to pH =8, is 73.2%. 

At this point it becomes clear that rGO is the best candidate for adsorbing Hg(II) ions (compared to GO) from aqueous solutions. Accordingly, in the remainder of the article, we focus only on the removal properties of rGO, considering three temperatures: 298, 313, and 333 K.

### 3.4. Adsorption Isotherms of rGO

**Figure 7:**
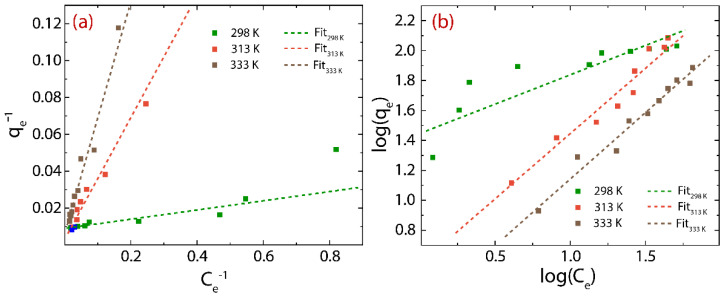
Adsorption isotherms: (**a**) Langmuir model and (**b**) Freundlich model.

The Hg(II) uptake capacity of rGO is evaluated by the adsorption isotherms (Figure 7) with a testing time of 20 min. 

The data points can be fitted using the approach of the Langmuir model (Figure 7a) as follows:

(9) $$q_{e}=\frac{q_{m}K_{L}C_{e}}{1+K_{L}C_{e}}\;$$

and the Freundlich model (Figure 7b) as follows:

(10) $$\log q_{e}=\log K_{F}+\frac{1}{n}\log C_{e}\;$$

where $q_{m}$ denotes the maximum adsorption capacity (mg g^−1^), $K_{L}$ denotes the Langmuir constant (L g^−1^), $K_{F}$ denotes the adsorption capacity and n denotes the heterogeneity of the adsorbent. The results are shown in Figure 7 as well as summarized in Table 4.

From the Langmuir model, the maximum adsorption capacity ($q_{m}$) also increases from 110.21 mg g^−1^ at 298 K to 255.04 mg g^−1^ at 333 K. The estimated $q_{m}$ values are higher than those of some recent reports such as GONR (33.02 mg g^−^^1^) [33], GO-TSC (231 mg g^−^^1^) [34], S-doped g-C3N4/LGO (46 mg g^−^^1^) [35], and HT-rGO-N (75.80 mg g^−^^1^) [37] (Table 5), suggesting that rGO is an interesting alternative compared to extra-functionalized/decorated oxidized graphenes. Additionally, rGO has an efficient adsorption time (20 min); for instance, S-GO seems to be more suitable for removing Hg(II) (3490 mg g^−^^1^) [32] since sulfur expands the affinity and specificity for Hg(II) ions. Nonetheless, its adsorption time (240 min) is 12 times slower than that of pristine rGO. 

From the Freundlich model, the values of n (1.44–0.26) found at different temperatures (298–333 K) demonstrate that the heterogeneity of rGO is minimal and tends to be homogeneous with increasing temperature [51]. The affinity of rGO for Hg(II) ions can be evidenced by the value of the $k_{F}$ parameter, where the respective results were found to be >0.1, which suggests, beyond the effect of temperature, a good affinity for Hg(II) ions [15]. 

### 3.5. Effect of Initial Concentration in rGO

**Figure 8:**
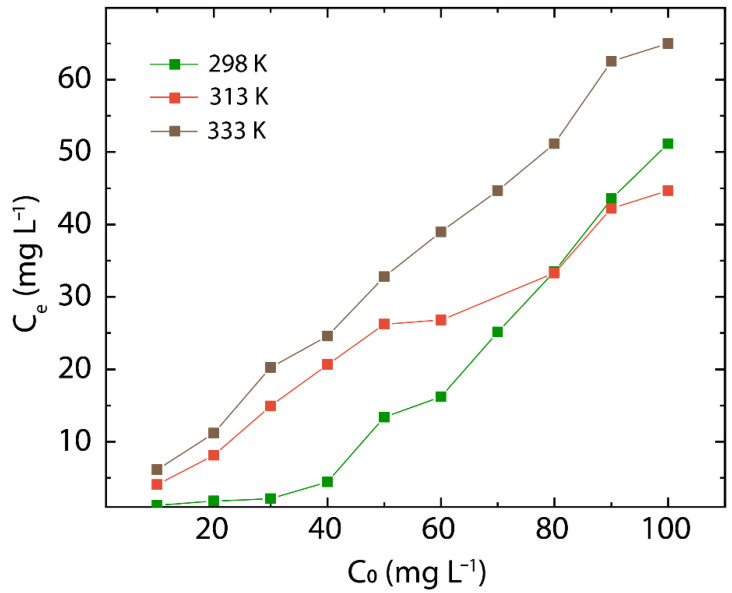
Effect of the initial concentration by analyzing $C_{e}$ as a function of $C_{0}$ at 298, 313, 333 K.

The equilibrium concentration is analyzed at 298, 313, and 333 K, which increases linearly with the initial concentration of Hg(II) in the solution ($C_{0}$) (Figure 8). In particular, a linear trend is observed in the range from 10 to 90 mg L^−1^ at 313 and 333 K and from 40 to 100 mg L^−1^ at 298 K. 

At higher equilibrium concentrations (≥90 mg L^−1^), a deviation from linearity does occur at 313 K (red markers) and 333 K (brown markers). Remarkably, at low initial concentrations (≤10 mg L^−1^), the equilibrium concentrations at 298 K (green points) and 313 K (red points) fall in the ppb range.

**Figure 9:**
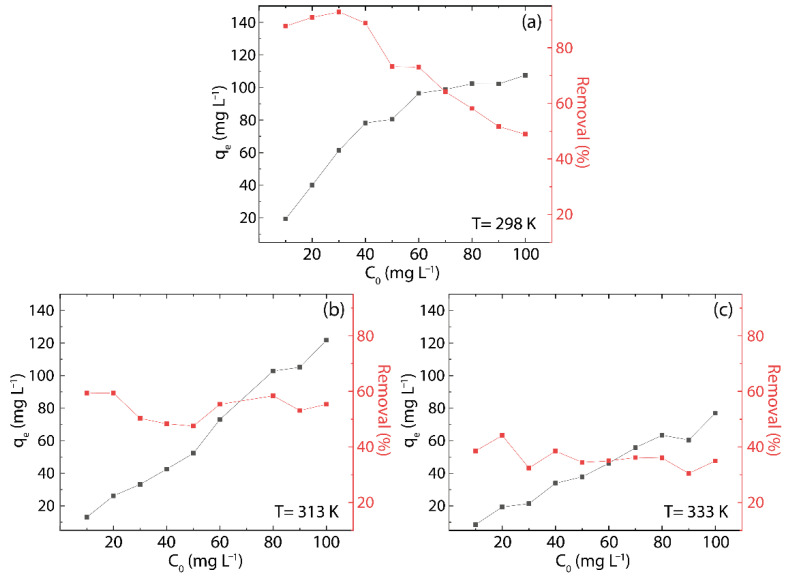
Adsorption capacity (black points) and removal percentage (red points) as a function of $C_{0}$ at (**a**) 298 K, (**b**) 313 K, and (**c**) 333 K.

Furthermore, the adsorption capacity of rGO increases quite linearly with the initial concentration of Hg(II) in the solution, almost in the range from 10 to 80 mg L^−1^ at 313 K (Figure 9b) and 333 K (Figure 9c), respectively. However, at 298 K (Figure 9a), the linearity is found in the range from 10 to 60 mg L^−1^, and for higher concentrations, a deviation is also observed, as reported in Figure 8. The latter outcomes suggest that rGO has a finite number of active sites for adsorption, increasing the value of $q_{e}$ whenever there is availability, and the saturation and maximum adsorption capacity ($q_{m}$) is reached as the active sites are covered during the adsorption process.

The efficacy of rGO appears to be independent of $C_{0}$, giving an average value of 54.1% at 313 K (Figure 9b, red markers) and 39.1% at 333 K (Figure 9c, red markers). In contrast, a strong dependency on $C_{0}$ is noted at 298 K, where the adsorption effectiveness decreases from 92.9% ($C_{0}=30$ mg L^−1^) down to 48.8% ($C_{0}=30$ mg L^−1^), resulting in an average value of 73.9% (Figure 9a, red markers).

### 3.6. Adsorption Thermodynamics in rGO

The Gibbs free energy ($\Delta G^{0}$), enthalpy change ($\Delta H^{0}$), and entropy change ($\Delta S^{0}$) are used to investigate the energy changes during the adsorption of Hg(II) on rGO [54,55,56]. These thermodynamics properties are estimated as follows:

(11) $$K_{e}=\frac{K_{L}\cdot M_{A}}{\gamma_{e}}$$

(12) $$\ln\;\frac{K_{e}}{\gamma_{e}}=\frac{\Delta S^{0}}{R}-\frac{\Delta H^{0}}{RT}≅\ln K_{e}$$

(13) $$\Delta G^{0}=-RT\ln K_{e}$$

where $K_{e}$ is the equilibrium constant (unitless), $K_{L}$ comes from the Langmuir constant, $\gamma_{e}$ is the activity coefficient (unitless), $M_{A}$ represents the molar weight of Hg [57], R is the gas constant, and T is the absolute temperature. 

Equations (11)–(13) can be used if the activity coefficient of the adsorbent is estimated from the Debye–Huckel limiting law or by considering the infinite dilute value of the equilibrium constant as γ≅1 [55]. 

**Figure 10:**
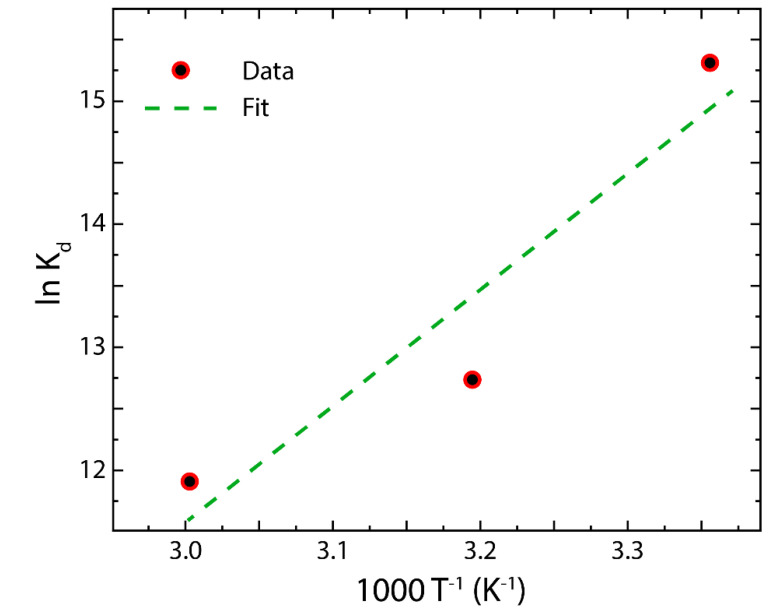
Analysis of the adsorption of Hg(II) on rGO by the Van ‘t Hoff approach.

The values of $\Delta H^{0}$ and $\Delta S^{0}$ are computed from the slope and intercept of the Van ‘t Hoff plot, i.e., $\ln\;K_{e}$ as a function of $T^{-1}$. The parameters of the Van ‘t Hoff approach are shown in Figure 10 and Table 6.

Negative values of $\Delta G^{0}$ point to the favorable nature of the adsorption of Hg(II) on rGO. Specifically, the $\Delta G^{0}$ values in the range from −39.43 to −32.30 kJ mol^−1^ (Table 6) suggest that the adsorption mechanism is ruled by a mixed physisorption–chemisorption process. Indeed, the adsorption process is called physisorption when the $\Delta G^{0}$ values are found in the range from 0 to −20 kJ mol^−1^, while if the values of $\Delta G^{0}$ are in the range from −80 to −400 kJ mol^−1^, the process is called chemisorption [58]. The missing region between these two ranges is somehow unclear [58,59]. Interestingly, as the temperature increases, the value of $\Delta G^{0}$ decreases by 16.49% at 313 K and by 18.08% at 333 K, demonstrating a weak interaction between rGO and Hg(II). The negative $\Delta H^{0}$ value of −98.31 kJ mol^−1^ supposes an exothermic process, advising a negative effect on the adsorption of Hg(II) ions, which results in higher adsorption at lower temperatures. The positive value of $\Delta S^{0}=$ 0.085 kJ mol^−1^ K^−1^ allows confirming the affinity of rGO for Hg(II) ions. The latter is in good agreement with IPD results.

### 3.7. Theoretical Insights on the Adsorption Mechanism by Density Functional Calculations 

**Figure 11:**
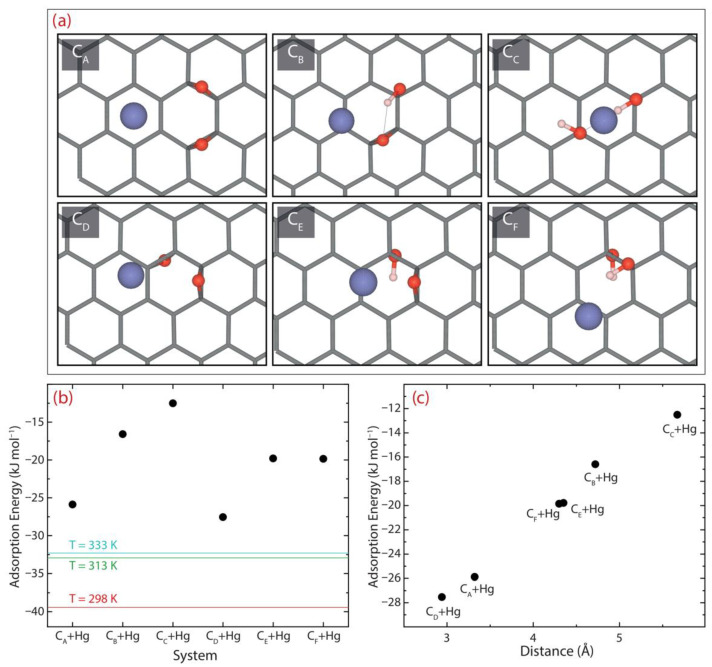
(**a**) Optimized pristine rGO structures obtained by DFT calculations and the six stable configurations of rGO covered by hydroxyl and epoxy groups interacting with the Hg atom. (**b**) The adsorption energy of the six interacting Hg-rGO systems. For comparison, the Gibbs free energy at different temperatures has been included (Table 6). (**c**) Adsorption energy as a function of the interaction distance between the Hg atom and rGO surface.

Although the chemical composition of oxidized graphenes remains unclear, it is commonly recognized that epoxy and hydroxyl groups are the principal functional groups found on the graphene surface [23]. Hence, in setting up the theoretical adsorption, six stable configurations were considered to recreate rGO models, namely C_A_, C_B_, C_C_, C_D_, C_E_, and C_F_ (Figure 11). Upon defining the different models, the adsorption process was simulated by engaging an Hg atom close to the oxygen functional groups and allowing the system to relax without restrictions. The optimized rGO models were then employed to estimate the adsorption energy ($E_{ads}$), which is calculated using the supermolecular approach, as follows:

(14) $$E_{ads}=E_{T\left(rGO+Hg\right)}-\left(E_{T\left(rGO\right)}+E_{T\left(Hg\right)}\right)\;$$

where $E_{T\left(rGO+Hg\right)}$ is the total energy of the interacting rGO+Hg system, $E_{T\left(rGO\right)}$ is the total energy of the rGO model, and $E_{T\left(Hg\right)}$ is the total energy of a single Hg atom. It is important to mention that the gas-phase calculations allowed the introduction of an explicit +2 charge into the system, i.e., Hg(II). The corresponding results are displayed in Figure 11 and Table 7.

Negative adsorption energies are found in all rGO+Hg systems, which shows an energetically favorable interaction for the adsorption of the Hg atom according to the results of adsorption thermodynamics (Figure 11b). Particularly, the theoretical adsorption energies showed that the most stable configurations are the C_A_ and C_D_ models with $E_{ads}=-25.88$ kJ mol^−1^ (−0.27 eV or −6.19 kcal mol^−1^) and $E_{ads}=-27.54$ kJ mol^−1^ (−0.29 eV or −6.85 kcal mol^−1^), respectively (Figure 11a and Table 7). Additionally, the distance from the rGO surface to the adsorbed Hg atom is found to be 3.32 Å for C_A_ and 2.94 Å for C_D_ (Figure 11c). These distances are very close to that observed between the graphene layers in graphite (3.32 Å [60]) and correspond to van der Waals-type interactions. This allows us to confirm that the adsorption mechanism of Hg(II) on rGO is described by a mixed physisorption–chemisorption process. The latter complements the results observed in the adsorption kinetics experiment.

Interestingly, the Hg atom is observed far away from the oxygen functional groups, with 3.65 Å for C_A_ and 3.42 Å for C_D_. These results suggest that Hg(II) ions prefer O-free zones, which, in such a scenario, are dominated by dispersive forces. Most importantly, it somehow suggests that the adsorption properties of rGO are more relevant compared to those of the GO (which is filled with oxygen functional groups but substantially lacks O-free zones). In addition, as the interaction distance between the rGO surface and the Hg atom increases, an energetically favorable linear adsorption is observed (Figure 11c). 

## 4. Conclusions

In summary, we have presented an eco-friendly protocol for preparing GO and rGO and the related morphological and spectroscopic characterizations. Most importantly, we have presented a comparative adsorption study of Hg(II) on either GO or rGO, which is analyzed in terms of the adsorption kinetics, adsorption isotherms, and adsorption thermodynamics. Our findings are complemented using quantum-mechanical calculations at the level of density functional theory. 

In particular, the change of GO into rGO is corroborated by SEM, TEM, EDS, Raman, FTIR, UV-Vis, and XRD analyses. Otherwise, the removal percentages of Hg(II) using GO or rGO from water were found to be ~28% and ~75%, respectively. These outcomes highlight the superior adsorbent properties of rGO compared to GO. From isotherm models, rGO shows a maximum adsorption capacity of 110.2 mg g^−1^ at 298 K and up to 255.0 mg g^−1^ at 333 K. DFT calculations propose a mixed physisorption–chemisorption process because the presence of oxygen functional groups favors the adsorption process of the Hg atom, but it also looks for oxygen-free zones. The theoretical predictions and data are in reasonable agreement. 

Our results are expected to be of immediate help for the proposal and study of non-extra-functionalized graphene-based materials for water treatment technologies.

## Figures and Tables

**Table 1:** Position and full-width at half maximum (FWHM) of peaks detected in GO and rGO in the region from 1000 to 2000 cm^−1^. The FWHM was obtained using Lorentzian fitting.

	D*—FWHM (cm^−1^)	D—FWHM (cm^−1^)	D**—FWHM (cm^−1^)	G—FWHM (cm^−1^)	D’—FWHM (cm^−1^)
**GO**	---	1348-122	1511-122	1576-58	1608-36
**rGO**	1122-79	1340-131	1522-127	1578-51	1605-38

**Table 2:** Parameters of pseudo-first-order and pseudo-second-order models are estimated at 298 K.

Parameters	Hg(II) on GO	Hg(II) on rGO
$q_{e}\left(exp\right)$ (mg g^−1^)	21.12	142.26
**Pseudo-first-order model**		
$q_{e}$ (mg g^−1^)	20.9 ± 0.7	143.7 ± 5.7
$k_{1}$ (min^−1^)	13.4 ± 0.4	0.194 ± 0.030
SSE	3.743	1826
*R* ^2^	0.985	0.931
RMSE	0.856	8.546
**Pseudo-second-order model**		
$q_{e}$ (mg g^−1^)	21.8 ± 0.7	151.3 ± 9.45
$k_{2}$ (g mg^−1^ min^−1^)	0.118 ± 0.001	0.002 ± 0.001
SSE	5.861	2480
*R* ^2^	0.990	0.949
RMSE	0.680	9.96

**Table 3:** Estimated parameters of the intraparticle diffusion (IPD) model at 298 K for the adsorption of Hg(II) on GO and rGO.

	Hg(II) on GO	Hg(II) on rGO
Parameters	Value	Value
$k_{p}$ (mg g^−1^ min^1/2^)	6.97 ± 0.67	7.82 ± 1.25
C (mg g^−1^)	8.65 ± 0.82	44.28 ± 7.75
$R_{i}$	0.586	0.411
R^2^	0.996	0.963

**Table 4:** Parameters of Langmuir and Freundlich isotherm models at 298, 313, and 333 K.

T (K)	Langmuir Model			Freundlich Model		
	$k_{L}$ (L g^−1^)	$q_{m}$ (mg g^−1^)	R^2^	$k_{F}$ (mg^(1-n)^ g^−1^ L^1/n^)	n	R^2^
298	4.71	110.21	0.933	0.592	1.44	0.936
313	1.56	217.34	0.947	0.872	0.57	0.979
333	0.58	255.04	0.964	0.885	0.26	0.978

**Table 5:** Comparative adsorption capacity and adsorption time for the removal of Hg(II) ions using oxidized graphenes.

Adsorbents	Adsorption Capacity (mg g^−1^)	Time (min)	Ref.
S-GO	3490	240	[32]
GONR (Hg and As)	33.02	12	[33]
GO-TSC	231	30	[34]
S-doped g-C_3_N4/LGO	46	120	[35]
GSH-NiFe_2_O_4_/GO	272.94	90	[36]
HT-rGO-N	75.80	10	[37]
${\mathrm{rGO}}_{298\;K}$ ${\mathrm{rGO}}_{313\;K}$ ${\mathrm{rGO}}_{333\;K}$	110.21 217.34 255.04	20	This work

**Table 6:** Parameters of the adsorption thermodynamics at 298, 313, and 333 K.

T (K)	$\Delta G^{0}$ (kJ mol^−1^)	$\Delta H^{0}$ (kJ mol^−1^)	$\Delta S^{0}$ (kJ mol^−1^ K^−1^)
298	−39.43		
313	−32.93	−98.31	0.085
333	−32.30		

**Table 7:** Adsorption energy predicted by Gaussian16 of the different rGO–Hg systems considered in the present work.

Adsorption Energy			
System	eV	kJ mol^−1^	kcal mol^−1^
C_A_ + Hg	−0.27	−25.88	−6.19
C_B_ + Hg	−0.17	−16.60	−3.97
C_C_ + Hg	−0.13	−12.51	−2.99
C_D_ + Hg	−0.29	−27.54	−6.85
C_E_ + Hg	−0.21	−19.79	−4.73
C_F_ + Hg	−0.21	−19.85	−4.74

## Data Availability

Not applicable.
